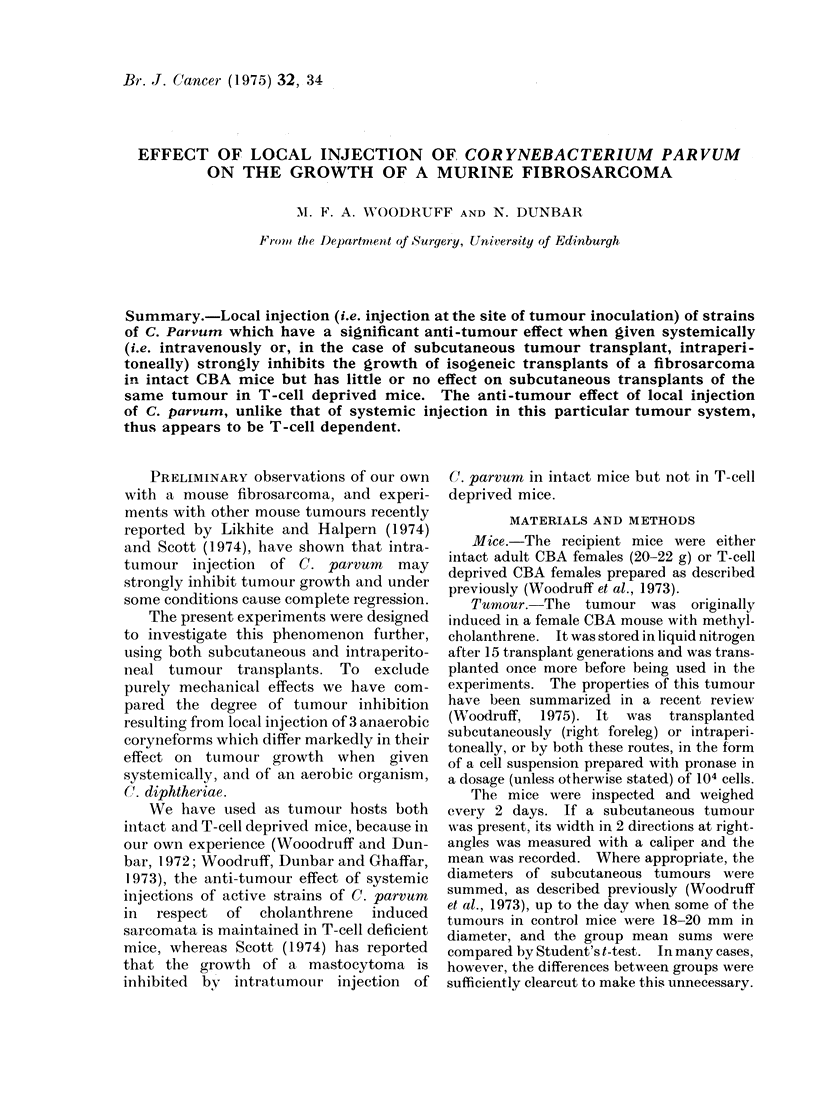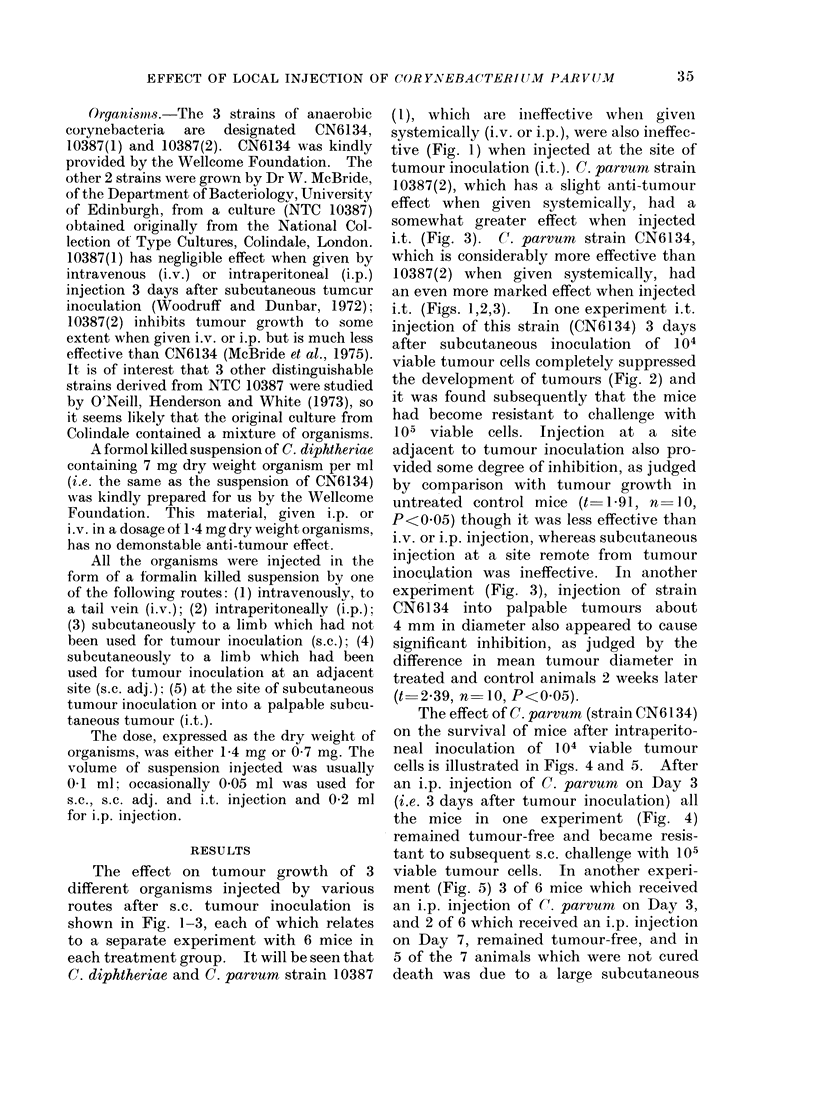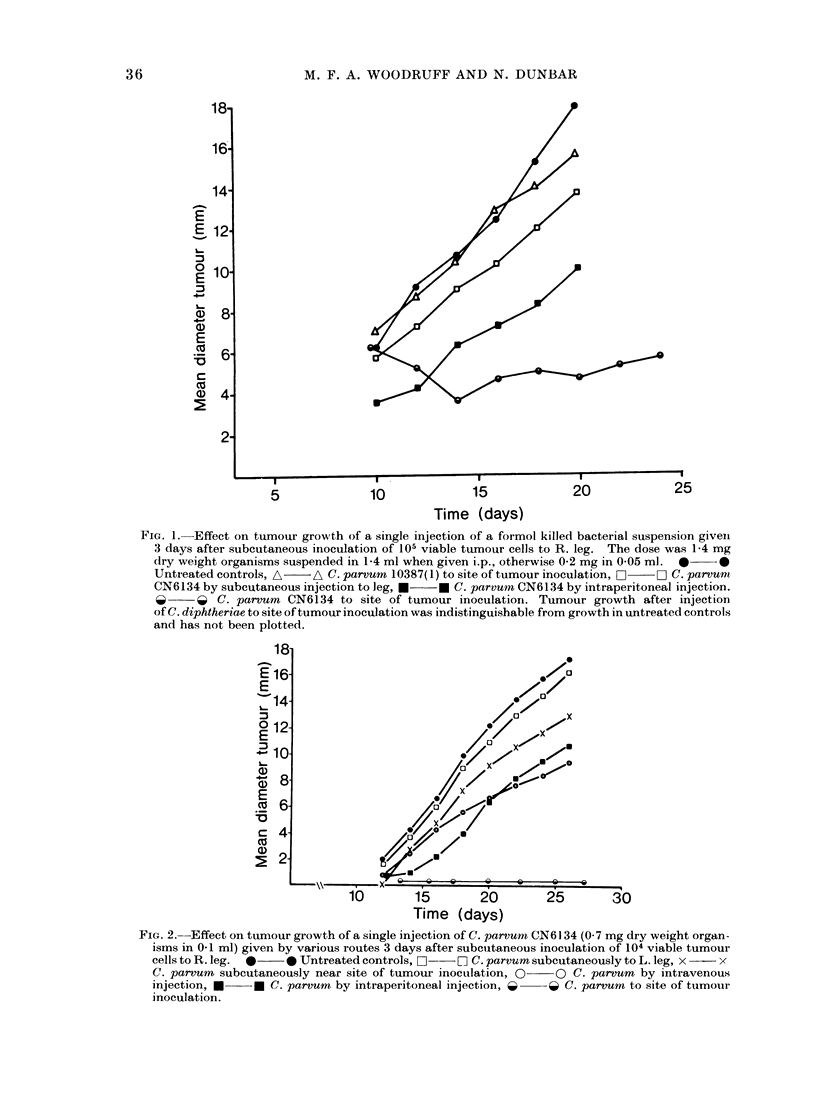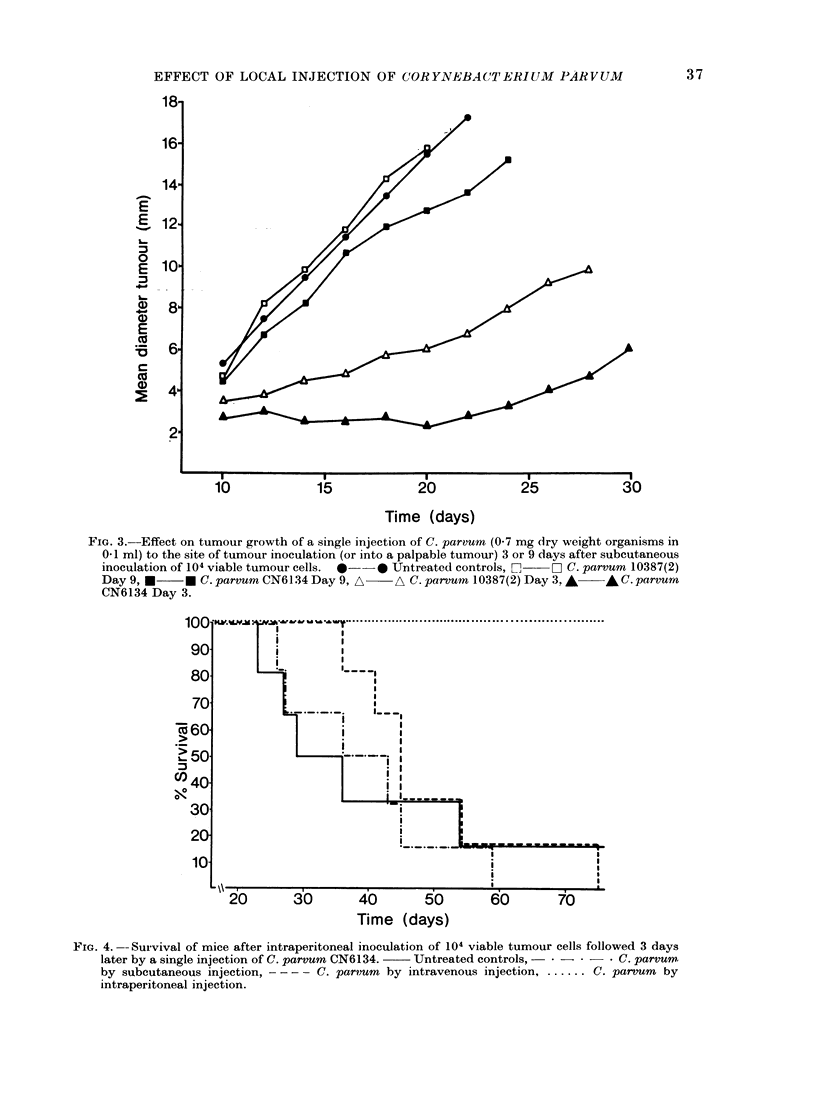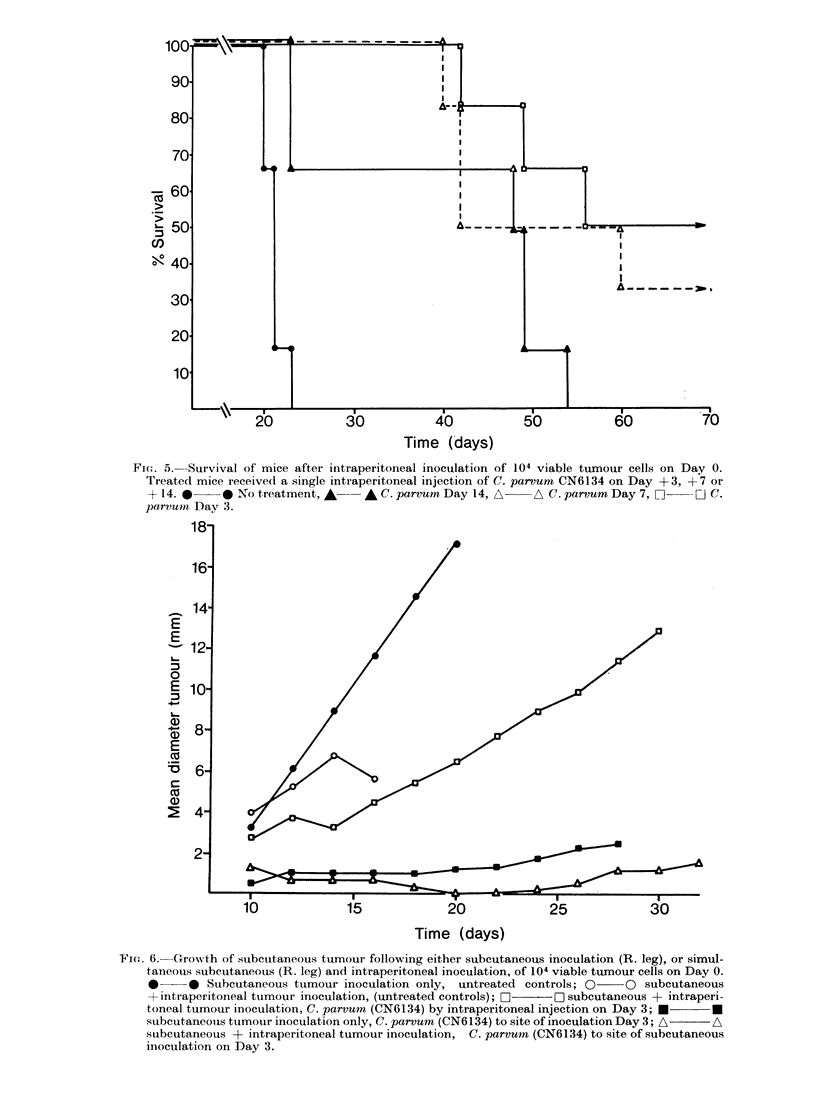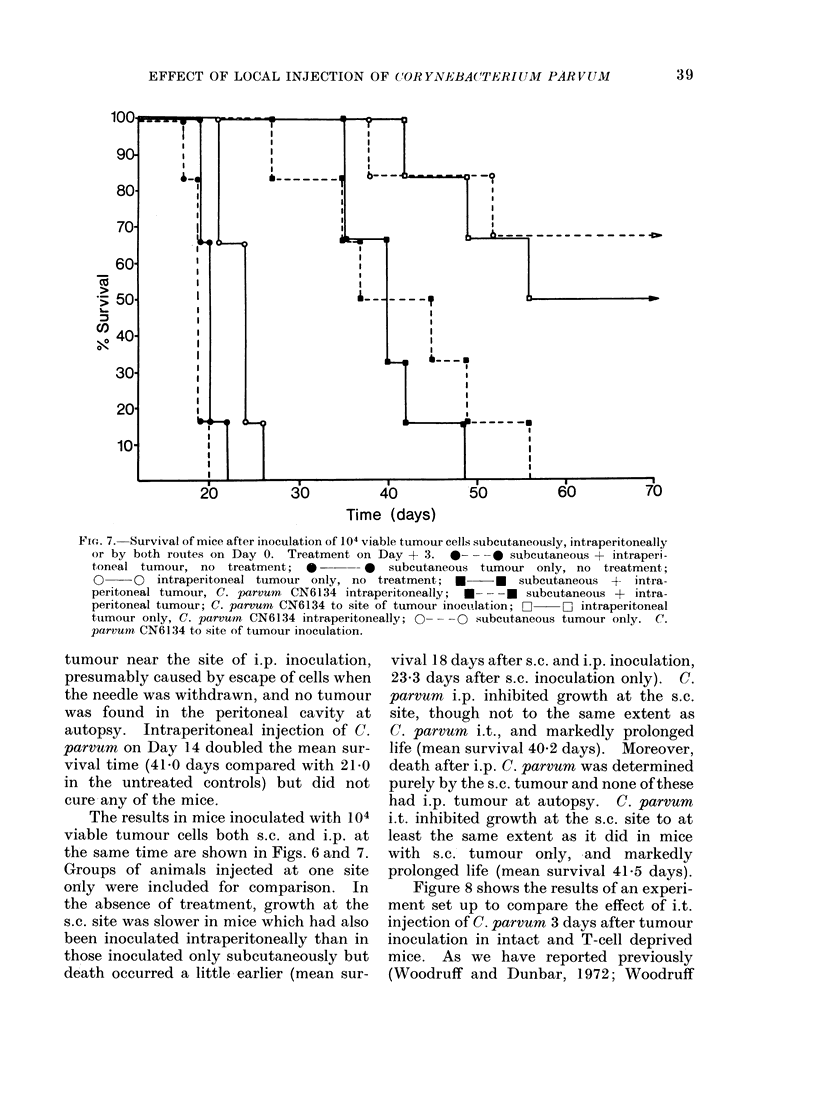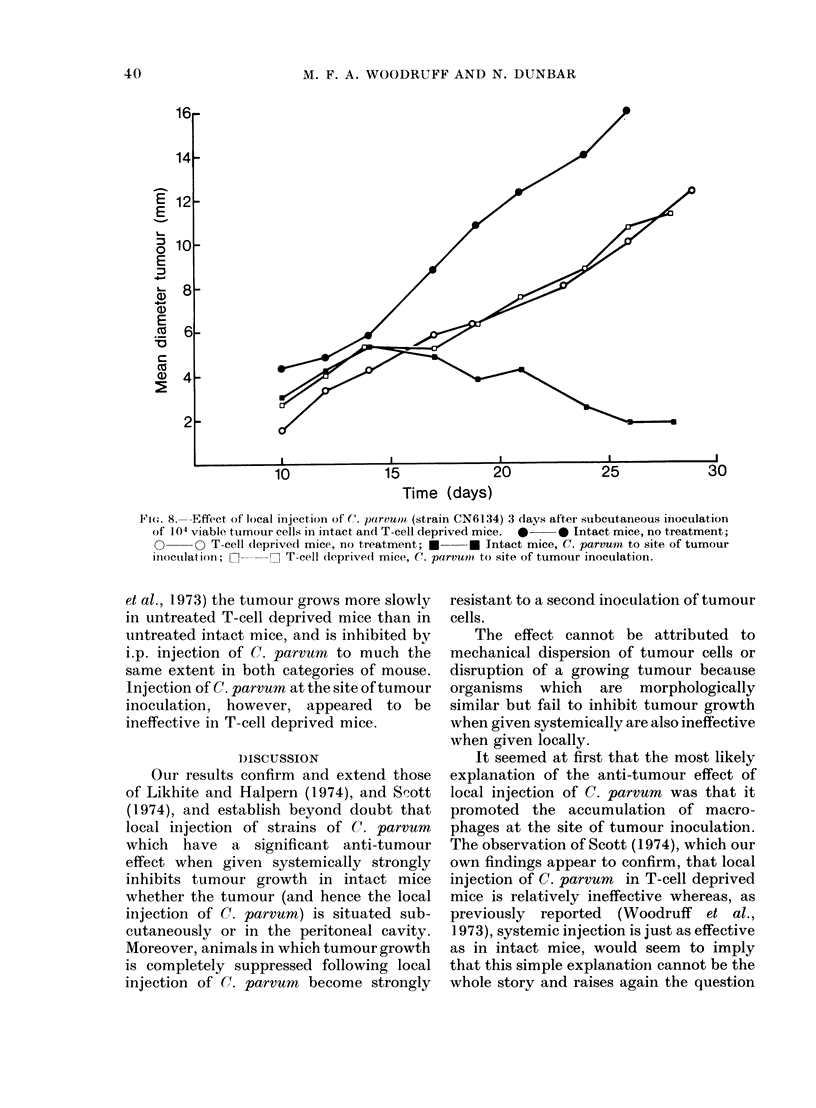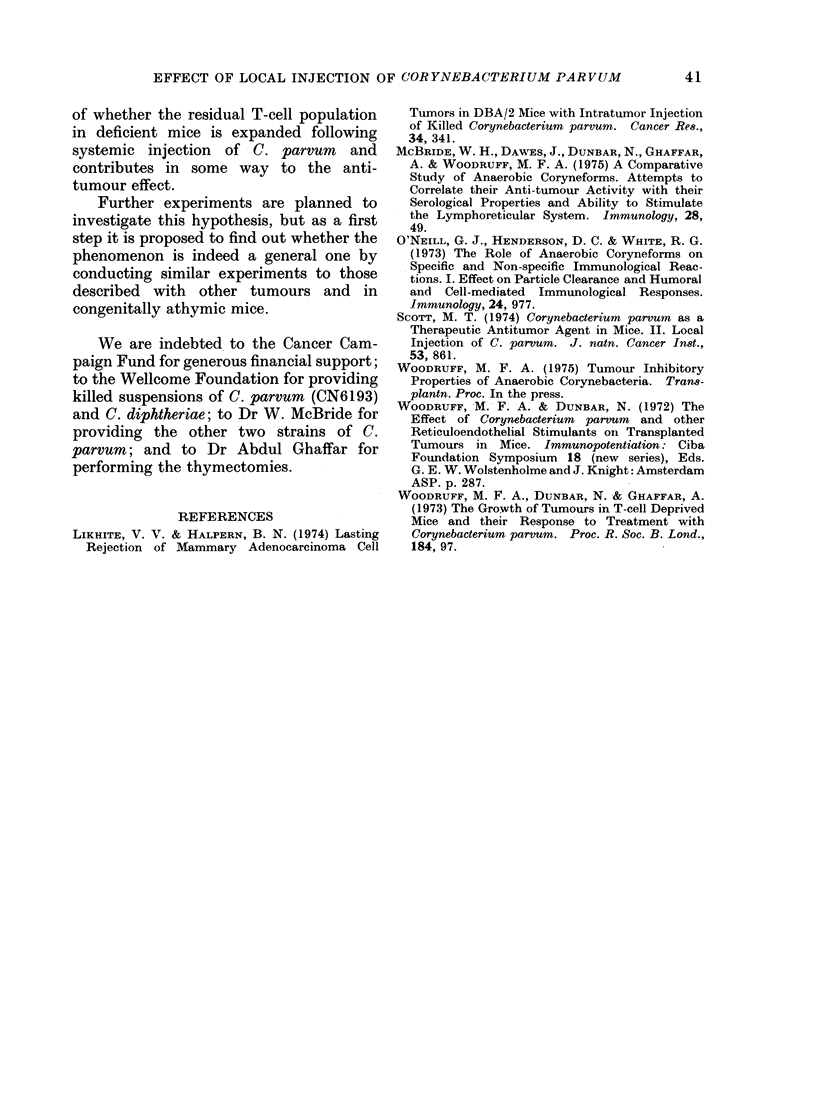# Effect of local injection of Corynebacterium parvum on the growth of a murine fibrosarcoma.

**DOI:** 10.1038/bjc.1975.131

**Published:** 1975-07

**Authors:** M. F. Woodruff, N. Dunbar

## Abstract

Local injection (i.e. injection at the site of tumour inoculation) of strains of C. Parvum which have a significant anti-tumour effect when given systemically (i.e. intravenously or, in the case of subcutaneous tumour transplant, intraperitoneally) strongly inhibits the growth of isogeneic transplants of a fibrosarcoma in intact CBA mice but has little or no effect on subcutaneous transplants of the same tumour in T-cell deprived mice. The anti-tumour effect of local injection of C. parvum, unlike that of systemic injection in this particular tumour system, thus appears to be T-cell dependent.


*Br. J. Cancer* (1975) **32**, 34

### EFFECT OF LOCAL INJECTION OF *CORYNEBACTERIUM PARVUM* ON THE GROWTH OF A MURINE FIBROSARCOMA

M. F. A. WOODRUFF AND N. DUNBAR

*From the Department of Surgery, University of Edinburgh*

**Summary.—Local injection (*i.e.* injection at the site of tumour inoculation) of strains of *C. Parvum* which have a significant anti-tumour effect when given systemically (*i.e.* intravenously or, in the case of subcutaneous tumour transplant, intraperitoneally) strongly inhibits the growth of isogeneic transplants of a fibrosarcoma in intact CBA mice but has little or no effect on subcutaneous transplants of the same tumour in T-cell deprived mice. The anti-tumour effect of local injection of *C. parvum*, unlike that of systemic injection in this particular tumour system, thus appears to be T-cell dependent.**

PRELIMINARY observations of our own with a mouse fibrosarcoma, and experiments with other mouse tumours recently reported by Likhite and Halpern (1974) and Scott (1974), have shown that intratumour injection of *C. parvum* may strongly inhibit tumour growth and under some conditions cause complete regression.

The present experiments were designed to investigate this phenomenon further, using both subcutaneous and intraperitoneal tumour transplants. To exclude purely mechanical effects we have compared the degree of tumour inhibition resulting from local injection of 3 anaerobic coryneforms which differ markedly in their effect on tumour growth when given systemically, and of an aerobic organism, *C. diphtheriae*.

We have used as tumour hosts both intact and T-cell deprived mice, because in our own experience (Wooodruff and Dunbar, 1972; Woodruff, Dunbar and Ghaffar, 1973), the anti-tumour effect of systemic injections of active strains of *C. parvum* in respect of cholanthrene induced sarcomata is maintained in T-cell deficient mice, whereas Scott (1974) has reported that the growth of a mastocytoma is inhibited by intratumour injection of *C. parvum* in intact mice but not in T-cell deprived mice.

### MATERIALS AND METHODS

*Mice*.—The recipient mice were either intact adult CBA females (20–22 g) or T-cell deprived CBA females prepared as described previously (Woodruff *et al.*, 1973).

*Tumour*.—The tumour was originally induced in a female CBA mouse with methylcholanthrene. It was stored in liquid nitrogen after 15 transplant generations and was transplanted once more before being used in the experiments. The properties of this tumour have been summarized in a recent review (Woodruff, 1975). It was transplanted subcutaneously (right foreleg) or intraperitoneally, or by both these routes, in the form of a cell suspension prepared with pronase in a dosage (unless otherwise stated) of $10^4$ cells.

The mice were inspected and weighed every 2 days. If a subcutaneous tumour was present, its width in 2 directions at right-angles was measured with a caliper and the mean was recorded. Where appropriate, the diameters of subcutaneous tumours were summed, as described previously (Woodruff *et al.*, 1973), up to the day when some of the tumours in control mice were 18–20 mm in diameter, and the group mean sums were compared by Student's *t*-test. In many cases, however, the differences between groups were sufficiently clearcut to make this unnecessary.

*Organisms.*—The 3 strains of anaerobic corynebacteria are designated CN6134, 10387(1) and 10387(2). CN6134 was kindly provided by the Wellcome Foundation. The other 2 strains were grown by Dr W. McBride, of the Department of Bacteriology, University of Edinburgh, from a culture (NTC 10387) obtained originally from the National Collection of Type Cultures, Colindale, London. 10387(1) has negligible effect when given by intravenous (i.v.) or intraperitoneal (i.p.) injection 3 days after subcutaneous tumcur inoculation (Woodruff and Dunbar, 1972); 10387(2) inhibits tumour growth to some extent when given i.v. or i.p. but is much less effective than CN6134 (McBride *et al.*, 1975). It is of interest that 3 other distinguishable strains derived from NTC 10387 were studied by O'Neill, Henderson and White (1973), so it seems likely that the original culture from Colindale contained a mixture of organisms.

A formol killed suspension of *C. diphtheriae* containing 7 mg dry weight organism per ml (*i.e.* the same as the suspension of CN6134) was kindly prepared for us by the Wellcome Foundation. This material, given i.p. or i.v. in a dosage of 1·4 mg dry weight organisms, has no demonstable anti-tumour effect.

All the organisms were injected in the form of a formalin killed suspension by one of the following routes: (1) intravenously, to a tail vein (i.v.); (2) intraperitoneally (i.p.); (3) subcutaneously to a limb which had not been used for tumour inoculation (s.c.); (4) subcutaneously to a limb which had been used for tumour inoculation at an adjacent site (s.c. adj.); (5) at the site of subcutaneous tumour inoculation or into a palpable subcutaneous tumour (i.t.).

The dose, expressed as the dry weight of organisms, was either 1·4 mg or 0·7 mg. The volume of suspension injected was usually 0·1 ml; occasionally 0·05 ml was used for s.c., s.c. adj. and i.t. injection and 0·2 ml for i.p. injection.

### RESULTS

The effect on tumour growth of 3 different organisms injected by various routes after s.c. tumour inoculation is shown in Fig. 1–3, each of which relates to a separate experiment with 6 mice in each treatment group. It will be seen that *C. diphtheriae* and *C. parvum* strain 10387 (1), which are ineffective when given systemically (i.v. or i.p.), were also ineffective (Fig. 1) when injected at the site of tumour inoculation (i.t.). *C. parvum* strain 10387(2), which has a slight anti-tumour effect when given systemically, had a somewhat greater effect when injected i.t. (Fig. 3). *C. parvum* strain CN6134, which is considerably more effective than 10387(2) when given systemically, had an even more marked effect when injected i.t. (Figs. 1,2,3). In one experiment i.t. injection of this strain (CN6134) 3 days after subcutaneous inoculation of $10^4$ viable tumour cells completely suppressed the development of tumours (Fig. 2) and it was found subsequently that the mice had become resistant to challenge with $10^5$ viable cells. Injection at a site adjacent to tumour inoculation also provided some degree of inhibition, as judged by comparison with tumour growth in untreated control mice ($t=1{\cdot}91$, $n=10$, $P<0{\cdot}05$) though it was less effective than i.v. or i.p. injection, whereas subcutaneous injection at a site remote from tumour inoculation was ineffective. In another experiment (Fig. 3), injection of strain CN6134 into palpable tumours about 4 mm in diameter also appeared to cause significant inhibition, as judged by the difference in mean tumour diameter in treated and control animals 2 weeks later ($t=2{\cdot}39$, $n=10$, $P<0{\cdot}05$).

The effect of *C. parvum* (strain CN6134) on the survival of mice after intraperitoneal inoculation of $10^4$ viable tumour cells is illustrated in Figs. 4 and 5. After an i.p. injection of *C. parvum* on Day 3 (*i.e.* 3 days after tumour inoculation) all the mice in one experiment (Fig. 4) remained tumour-free and became resistant to subsequent s.c. challenge with $10^5$ viable tumour cells. In another experiment (Fig. 5) 3 of 6 mice which received an i.p. injection of *C. parvum* on Day 3, and 2 of 6 which received an i.p. injection on Day 7, remained tumour-free, and in 5 of the 7 animals which were not cured death was due to a large subcutaneous

FIG. 1.—Effect on tumour growth of a single injection of a formol killed bacterial suspension given 3 days after subcutaneous inoculation of $10^5$ viable tumour cells to R. leg. The dose was 1·4 mg dry weight organisms suspended in 1·4 ml when given i.p., otherwise 0·2 mg in 0·05 ml. ●——● Untreated controls, △——△ *C. parvum* 10387(1) to site of tumour inoculation, □——□ *C. parvum* CN6134 by subcutaneous injection to leg, ■——■ *C. parvum* CN6134 by intraperitoneal injection. ◒——◒ *C. parvum* CN6134 to site of tumour inoculation. Tumour growth after injection of *C. diphtheriae* to site of tumour inoculation was indistinguishable from growth in untreated controls and has not been plotted.

FIG. 2.—Effect on tumour growth of a single injection of *C. parvum* CN6134 (0·7 mg dry weight organisms in 0·1 ml) given by various routes 3 days after subcutaneous inoculation of $10^4$ viable tumour cells to R. leg. ●——● Untreated controls, □——□ *C. parvum* subcutaneously to L. leg, ×——× *C. parvum* subcutaneously near site of tumour inoculation, ○——○ *C. parvum* by intravenous injection, ■——■ *C. parvum* by intraperitoneal injection, ◒——◒ *C. parvum* to site of tumour inoculation.

FIG. 3.—Effect on tumour growth of a single injection of *C. parvum* (0·7 mg dry weight organisms in 0·1 ml) to the site of tumour inoculation (or into a palpable tumour) 3 or 9 days after subcutaneous inoculation of $10^4$ viable tumour cells. ●——● Untreated controls, □——□ *C. parvum* 10387(2) Day 9, ■——■ *C. parvum* CN6134 Day 9, △——△ *C. parvum* 10387(2) Day 3, ▲——▲ *C. parvum* CN6134 Day 3.

FIG. 4.—Survival of mice after intraperitoneal inoculation of $10^4$ viable tumour cells followed 3 days later by a single injection of *C. parvum* CN6134. —— Untreated controls, — · — · — · *C. parvum* by subcutaneous injection, – – – – *C. parvum* by intravenous injection, ...... *C. parvum* by intraperitoneal injection.

FIG. 5.—Survival of mice after intraperitoneal inoculation of $10^4$ viable tumour cells on Day 0. Treated mice received a single intraperitoneal injection of *C. parvum* CN6134 on Day +3, +7 or +14. ●——● No treatment, ▲——▲ *C. parvum* Day 14, △——△ *C. parvum* Day 7, □——□ *C. parvum* Day 3.

FIG. 6.—Growth of subcutaneous tumour following either subcutaneous inoculation (R. leg), or simultaneous subcutaneous (R. leg) and intraperitoneal inoculation, of $10^4$ viable tumour cells on Day 0. ●——● Subcutaneous tumour inoculation only, untreated controls; ○——○ subcutaneous + intraperitoneal tumour inoculation, (untreated controls); □——□ subcutaneous + intraperitoneal tumour inoculation, *C. parvum* (CN6134) by intraperitoneal injection on Day 3; ■——■ subcutaneous tumour inoculation only, *C. parvum* (CN6134) to site of inoculation Day 3; △——△ subcutaneous + intraperitoneal tumour inoculation, *C. parvum* (CN6134) to site of subcutaneous inoculation on Day 3.

FIG. 7.—Survival of mice after inoculation of $10^4$ viable tumour cells subcutaneously, intraperitoneally or by both routes on Day 0. Treatment on Day + 3. ●– – –● subcutaneous + intraperitoneal tumour, no treatment; ●——● subcutaneous tumour only, no treatment; ○——○ intraperitoneal tumour only, no treatment; ■——■ subcutaneous + intraperitoneal tumour, *C. parvum* CN6134 intraperitoneally; ■– – –■ subcutaneous + intraperitoneal tumour; *C. parvum* CN6134 to site of tumour inoculation; □——□ intraperitoneal tumour only, *C. parvum* CN6134 intraperitoneally; ○– – –○ subcutaneous tumour only. *C. parvum* CN6134 to site of tumour inoculation.

tumour near the site of i.p. inoculation, presumably caused by escape of cells when the needle was withdrawn, and no tumour was found in the peritoneal cavity at autopsy. Intraperitoneal injection of *C. parvum* on Day 14 doubled the mean survival time (41·0 days compared with 21·0 in the untreated controls) but did not cure any of the mice.

The results in mice inoculated with $10^4$ viable tumour cells both s.c. and i.p. at the same time are shown in Figs. 6 and 7. Groups of animals injected at one site only were included for comparison. In the absence of treatment, growth at the s.c. site was slower in mice which had also been inoculated intraperitoneally than in those inoculated only subcutaneously but death occurred a little earlier (mean survival 18 days after s.c. and i.p. inoculation, 23·3 days after s.c. inoculation only). *C. parvum* i.p. inhibited growth at the s.c. site, though not to the same extent as *C. parvum* i.t., and markedly prolonged life (mean survival 40·2 days). Moreover, death after i.p. *C. parvum* was determined purely by the s.c. tumour and none of these had i.p. tumour at autopsy. *C. parvum* i.t. inhibited growth at the s.c. site to at least the same extent as it did in mice with s.c. tumour only, and markedly prolonged life (mean survival 41·5 days).

Figure 8 shows the results of an experiment set up to compare the effect of i.t. injection of *C. parvum* 3 days after tumour inoculation in intact and T-cell deprived mice. As we have reported previously (Woodruff and Dunbar, 1972; Woodruff

FIG. 8.—Effect of local injection of *C. parvum* (strain CN6134) 3 days after subcutaneous inoculation of $10^4$ viable tumour cells in intact and T-cell deprived mice. ●——● Intact mice, no treatment; ○——○ T-cell deprived mice, no treatment; ■——■ Intact mice, *C. parvum* to site of tumour inoculation; □——□ T-cell deprived mice, *C. parvum* to site of tumour inoculation.

*et al.*, 1973) the tumour grows more slowly in untreated T-cell deprived mice than in untreated intact mice, and is inhibited by i.p. injection of *C. parvum* to much the same extent in both categories of mouse. Injection of *C. parvum* at the site of tumour inoculation, however, appeared to be ineffective in T-cell deprived mice.

### DISCUSSION

Our results confirm and extend those of Likhite and Halpern (1974), and Scott (1974), and establish beyond doubt that local injection of strains of *C. parvum* which have a significant anti-tumour effect when given systemically strongly inhibits tumour growth in intact mice whether the tumour (and hence the local injection of *C. parvum*) is situated subcutaneously or in the peritoneal cavity. Moreover, animals in which tumour growth is completely suppressed following local injection of *C. parvum* become strongly resistant to a second inoculation of tumour cells.

The effect cannot be attributed to mechanical dispersion of tumour cells or disruption of a growing tumour because organisms which are morphologically similar but fail to inhibit tumour growth when given systemically are also ineffective when given locally.

It seemed at first that the most likely explanation of the anti-tumour effect of local injection of *C. parvum* was that it promoted the accumulation of macrophages at the site of tumour inoculation. The observation of Scott (1974), which our own findings appear to confirm, that local injection of *C. parvum* in T-cell deprived mice is relatively ineffective whereas, as previously reported (Woodruff *et al.*, 1973), systemic injection is just as effective as in intact mice, would seem to imply that this simple explanation cannot be the whole story and raises again the question of whether the residual T-cell population in deficient mice is expanded following systemic injection of *C. parvum* and contributes in some way to the anti-tumour effect.

Further experiments are planned to investigate this hypothesis, but as a first step it is proposed to find out whether the phenomenon is indeed a general one by conducting similar experiments to those described with other tumours and in congenitally athymic mice.

We are indebted to the Cancer Campaign Fund for generous financial support; to the Wellcome Foundation for providing killed suspensions of *C. parvum* (CN6193) and *C. diphtheriae*; to Dr W. McBride for providing the other two strains of *C. parvum*; and to Dr Abdul Ghaffar for performing the thymectomies.

### REFERENCES

LIKHITE, V. V. & HALPERN, B. N. (1974) Lasting Rejection of Mammary Adenocarcinoma Cell Tumors in DBA/2 Mice with Intratumor Injection of Killed *Corynebacterium parvum*. *Cancer Res.*, **34**, 341.

MCBRIDE, W. H., DAWES, J., DUNBAR, N., GHAFFAR, A. & WOODRUFF, M. F. A. (1975) A Comparative Study of Anaerobic Coryneforms. Attempts to Correlate their Anti-tumour Activity with their Serological Properties and Ability to Stimulate the Lymphoreticular System. *Immunology*, **28**, 49.

O'NEILL, G. J., HENDERSON, D. C. & WHITE, R. G. (1973) The Role of Anaerobic Coryneforms on Specific and Non-specific Immunological Reactions. I. Effect on Particle Clearance and Humoral and Cell-mediated Immunological Responses. *Immunology*, **24**, 977.

SCOTT, M. T. (1974) *Corynebacterium parvum* as a Therapeutic Antitumor Agent in Mice. II. Local Injection of *C. parvum*. *J. natn. Cancer Inst.*, **53**, 861.

WOODRUFF, M. F. A. (1975) Tumour Inhibitory Properties of Anaerobic Corynebacteria. *Transplantn. Proc.* In the press.

WOODRUFF, M. F. A. & DUNBAR, N. (1972) The Effect of *Corynebacterium parvum* and other Reticuloendothelial Stimulants on Transplanted Tumours in Mice. *Immunopotentiation:* Ciba Foundation Symposium **18** (new series), Eds. G. E. W. Wolstenholme and J. Knight: Amsterdam ASP. p. 287.

WOODRUFF, M. F. A., DUNBAR, N. & GHAFFAR, A. (1973) The Growth of Tumours in T-cell Deprived Mice and their Response to Treatment with *Corynebacterium parvum*. *Proc. R. Soc. B. Lond.*, **184**, 97.